# Breastfeeding Experience among Mothers during the COVID-19 Pandemic

**DOI:** 10.3390/ijerph19084535

**Published:** 2022-04-09

**Authors:** Hanan Badr, Salmah Alghamdi

**Affiliations:** Maternity and Child Department, Faculty of Nursing, King Abdulaziz University, Jeddah 21589, Saudi Arabia; saalghamdi6@kau.edu.sa

**Keywords:** pandemic, COVID-19, breastfeeding, qualitative, experience, support

## Abstract

When health experts declared COVID-19 to be a global pandemic, they recognized the virus as a major environmental factor that could affect the practice of breastfeeding. A few studies focused on the effect of COVID-19 on mothers who gave birth during the pandemic. The purpose of this study is to explore the experience of Saudi Arabian breastfeeding mothers during the COVID-19 pandemic. This study used a descriptive phenomenology qualitative design and a convenience sample of 18 mothers who breastfed their children beginning in March 2020. Data were collected through semi-structured, open-ended phone interviews and analyzed using thematic analysis. The mothers were between 27 and 36 years old, and most of them had previous breastfeeding experience. Four main themes emerged regarding breastfeeding experiences during the COVID-19 pandemic: breastfeeding experience (positive and negative), support, facilitators, and challenges. Most mothers felt their experience with breastfeeding during the pandemic encouraged them to continue. It is important to reassure and educate breastfeeding mothers about the nature of COVID-19 and its mode of transmission. The findings from this study lay the foundation for future research to support the practice of breastfeeding and overcome the challenges that arose during the pandemic.

## 1. Introduction

Exclusive breastfeeding is the most complete form of infant nutrition; it establishes a healthy foundation for infant growth and development. The World Health Organization [1] (WHO) recommends exclusive breastfeeding for newborns starting from the first hour after birth until at least 6 months, and ideally breastfeeding should be continued until the child is 2 years old [1].

The WHO declared the outbreak of COVID-19 to be a global pandemic in March 2020 [1]. Like all countries around the world, Saudi Arabia was affected by COVID-19. From March 2020 until February 2021, there were almost 370,000 cases in Saudi Arabia, and this number increased cumulatively every day [2]. The length and experience of breastfeeding could have been affected during this time because of the COVID-19 pandemic [3].

Breastfeeding has several well-documented benefits for both infants and mothers. For mothers, breastfeeding reduces the risk of breast and ovarian cancer. For infants, breastfeeding provides antibodies that protect against organisms that cause diseases, prevent obesity, and improve intelligence [4]. Therefore, it is crucial to support the practice of breastfeeding.

Breastfeeding is a complex process that is influenced by several personal and environmental factors. Personal psychosocial variables, such as support and financial, cultural, and religious factors, may influence breastfeeding initiation and continuation [5,6]. The global COVID-19 pandemic was a major environmental factor that may have affected the practice of breastfeeding. A recent literature review revealed increased marketing and distribution of infant formula in emergencies either caused by conflicts or natural disasters [7], which may also have influenced the initiation and continuation of breastfeeding.

Several cases of COVID-19 were identified among pregnant women [8,9], with a noticeable increase in the rates of cesarean deliveries. Hospitals adjusted their policies and procedures for labor and delivery to maintain the safety of mothers and their infants, including decreasing visitors and stay periods. Moreover, there were many governmental orders issued during the pandemic to control the spread of the virus. These orders included partial and complete curfews in Saudi Arabia from March to July 2020 and a complete lockdown for some cities and neighborhoods for almost 3 months. All places of entertainment, restaurants, and public beaches were completely closed, and social distancing was required. These orders affected communities financially, socially, and psychologically [10]; however, only a few studies have focused on the effect on mothers who gave birth during the pandemic.

The WHO recommends breastfeeding and rooming-in whether or not the mothers or their infants have a suspected or confirmed COVID-19 infection [1]. Studies have shown that the expressed breast milk of mothers infected with COVID-19 is safe for their newborns [8,11]. However, some researchers did not recommend direct breastfeeding and suggested isolating infected mothers with SARS-CoV-2 from their infants for 14 days [12]. Among mothers with confirmed COVID-19 infections, bonding, rooming-in, and breastfeeding were of great concern.

The rate of breastfeeding initiation in the Kingdom of Saudi Arabia (KSA) has been consistently high, at over 90% [13,14,15]. However, the mode of delivery (vaginal delivery or cesarean) is a significant predictor of breastfeeding initiation [13]. Given increased rates of cesarean sections among mothers with positive COVID-19 infections, the initiation and continuation of breastfeeding must be supported.

A recent study showed a significantly lower exclusive breastfeeding rate among women who gave birth during the COVID-19 lockdown than mothers who gave birth the previous year [16]. Mothers reported stress, isolation, and anxiety in their breastfeeding experience during the pandemic [6], and these factors could be barriers to breastfeeding. Most of the breastfeeding mothers in the UK stopped breastfeeding because they could not find enough support during the lockdown and felt isolated [3]. Some mothers sought support to continue breastfeeding during the pandemic [6].

The COVID-19 pandemic affected the practice of breastfeeding because its influence is personal, psychological, environmental, and social. A few articles have focused on appropriate care for pregnant and postpartum women with positive COVID-19 diagnoses [17,18,19]. However, the breastfeeding experiences of mothers during the pandemic remain widely unexplored in the KSA. Thus, the purpose of this study is to explore the experiences of Saudi Arabian breastfeeding mothers during the COVID-19 pandemic.

## 2. Materials and Methods

### 2.1. Design

The study design used in this research was a descriptive, phenomenological, qualitative design. It was used to collect detailed information about the mothers’ experiences with breastfeeding during the COVID-19 pandemic. Using a qualitative research design increases understanding of a specific perception from the participants’ point of view. In addition, this method provides a rich narrative about the phenomena that helped uncover the entire picture [20]. According to Streubert and Carpenter [20], descriptive phenomenology helps researchers describe and provide in-depth information about the mothers’ lived experience of a phenomenon [20].

### 2.2. Sample and Recruitment

We chose a convenience sample of mothers who breastfed their children since March 2020 (the start date of the lockdown period) and were willing to participate in the study. The suggested sample size was 5–25 participants, based on data saturation, and the inclusion criteria were the following: (1) age 18 years and older, (2) Arabic speakers, (3) mothers who breastfed their newborns since March 2020, and (4) mothers who resided in Saudi Arabia. In this study, we reached information saturation with 18 participants; all participants completed the survey.

To recruit the participants, the researchers created a flyer to be posted on social media (WhatsApp, Facebook, and Twitter) to be distributed among the population. The flyer included the study aim, the target population, and contact information, so the mothers wanting to participate could contact the researchers. Any participant willing to participate could call the principal investigator to determine whether she met the inclusion criteria.

### 2.3. Data Collection

We conducted semi-structured, open-ended interviews by telephone to explore the mothers’ breastfeeding challenges and facilitators during COVID-19 in KSA. Each mother was interviewed individually, and the interview was recorded using an MP3 voice recorder. Each interview lasted between 20 and 25 min.

After the participants contacted the principal researcher, and the researcher confirmed that a participant met the inclusion criteria, a date was set to complete the phone interview. Each participant’s verbal consent was obtained before starting the recording. Afterward, the mother’s demographic information was gathered, and the interview was started. The researchers began by asking the mothers about their experiences with breastfeeding during COVID-19. They were asked, “Tell me about your experience with breastfeeding during COVID-19”. Then, more probing questions were asked to gather more information about the mothers, and feedback was sought to ensure that the researchers clearly understood the mothers’ responses. Some of the probing questions included the following: How do you describe their experience of breastfeeding during the COVID-19 pandemic? What are the facilitators that affected breastfeeding during COVID-19? What are the challenges that affected breastfeeding during COVID-19? What are your ideas about the differences between the breastfeeding experience before and after the COVID-19 pandemic? What are your suggestions to help breastfeeding mothers continue breastfeeding during the COVID-19 pandemic or during any future pandemics?

### 2.4. Ethical Considerations

Ethical approval was obtained from the faculty of nursing at King Abdulaziz University (NREC Serial No: Ref No 2F. 39). All participants provided verbal consent and were informed that they had the right to withdraw from the study at any time. They also had the right not to answer any question that made them uncomfortable. Researchers maintained the confidentiality of participants’ information throughout the process.

### 2.5. Data Analysis

Data collection and analysis were conducted simultaneously. In our research, the six steps of thematic analysis suggested by Polit and Beck were used [21]. All the recordings were transcribed and then the researchers reviewed all the transcriptions against the recordings to ensure the transcriptions were correct. After that, all their transcriptions were sent to the participants for review to ensure the written information in the script was what the participant intended to provide. Both researchers then reviewed the transcripts line by line to highlight the main codes related to our research. A table for all the codes was created to organize them into themes.

The trustworthiness of the qualitative research was assured by sending each participant their transcription to confirm the accuracy of the written information. Both researchers had previous experience conducting qualitative research, and they worked together during data collection and analysis to ensure different perspectives during the process.

## 3. Results

In this study, 18 mothers were recruited. Their ages ranged from 27 to 36 years old. All of the mothers were married. Of the total participants, 47% (*n* = 8), were housewives, 53% (*n* = 9) were working mothers, and only one participant was a university student. Regarding income, 35% (*n* = 6) of participants had a monthly income between 3000 and 6999 Saudi Riyals (SR), 41% (*n* = 7) had an income ranging from 7000 to 9999 SR, and 24% (*n* = 5) of participants had an income of more than 10,000 SR. Other demographic information is presented in Table 1, including obstetric history, breastfeeding history, and the incidence of contracting COVID-19 during the breastfeeding period.

### 3.1. Findings Themes

In our study, we found that breastfeeding during COVID-19 has four main themes: the breastfeeding experience (positive and negative), support and resources that helped mothers continue breastfeeding during COVID-19, facilitators of breastfeeding, and challenges preventing mothers from continuing to breastfeed (Table 2).

#### 3.1.1. The Breastfeeding Experience

The mothers were asked about their overall experiences with breastfeeding their current child. Follow-up questions for multipara mothers included whether their breastfeeding experience was different from before COVID-19. Even though the breastfeeding experiences varied between positive and negative, there were some mothers who mentioned that there was no difference between breastfeeding before and after COVID-19. Some of the mothers stated, *“Nothing has changed.”*

##### Positive Experiences

Most of the mothers reported similar reasons for having positive experiences with breastfeeding. They said that breastfeeding increased the bond between the mother and the newborn. One of the mothers stated, *“I feel that Corona is better in terms of breastfeeding. Because there are no visits and there are no guests who come for a while, I spent more time with my daughter.”* Another mother stated, *“It was easy for me to breastfeed. There were not many obstacles. It is true my baby was so attached to me because he was all the time with me at home.”*

Most of the mothers were breastfeeding their children to strengthen the newborns’ immunity (especially during the COVID-19 pandemic). One of the mothers stated, *“I care about my child, I know that breastfeeding will increase his immunity, especially during this time.”*

Breastfeeding is easier than bottle feeding because there is no need to pump or prepare bottles. One of the mothers stated, *“With Corona, the situation is easier. I work from home. Being next to the baby made it easier, as there is no need for using a breast pump during this period.”* Another mother stated, *“At the time of the curfew, we sat at home, so I was breastfeeding him directly. I didn’t need a cup or anything.”*

##### Negative Experiences

The participating mothers mentioned different reasons for having negative experiences with breastfeeding, including difficulty, commitment, pain, effort, worry, and fear of contracting the coronavirus. One of the mothers stated, *“It is hard to breastfeed during the night, especially while I was sleeping.”* Another mother stated, *” Frankly, I think of weaning my daughter because I am tired; I have become a pacifier, and I cannot sleep—it needs effort, and I am exhausted.”* Regarding worries about contracting the coronavirus, one of the mothers stated, *“I mean, at one time, I thought that I had corona because suddenly I had fever and pain, so I was worried how the baby would breastfeed.”* Regarding the pain, one of the mothers stated, *“It was a difficult time. I had severe pain in my breast. I had to breastfed her by bottle until the pain relieved.”*

#### 3.1.2. Support and Resources

The interviewed mothers pointed out the importance of support in their breastfeeding experience during COVID-19. The sources of support varied among the participants. Most mentioned their husbands the most supportive family member. The following sentiment was commonly reported by the participants: *“My husband was there for me and helped me in all circumstances.”* Another mother stated that her husband *“insisted on breastfeeding.”* However, one of the participating mothers in this study felt that her husband was unsupportive and was the reason for her discontinuing breastfeeding; she stated, *“My husband asks me to give formula feeding whenever the baby cries.”*

The second-most-mentioned supportive family member for participating mothers was their own mothers, followed by friends and colleagues. The following statement was echoed by various participants: “*My mom says, don’t worry, breastfeed her, till at least 6 months old.”* The following statement illustrates colleague support: *“It was my colleague who encouraged me to breastfeed, and to pump and store my breast milk to use while I am back to work.”*

Moreover, the presence of a lactation consultant before hospital discharge was described as supportive for breastfeeding; however, not all participating mothers had a breastfeeding session with a lactation consultant at the hospital. One of the mothers described her experience with the lactation consultant by saying, *“She helped me a bit.”* Some mothers described social media as having supportive resources where they could read and learn about breastfeeding advantages. For example, *“Whenever my enthusiasm decreased, I would go and read about its benefits and how does it affect the brain and the child’s immunity and digestive systems; it is very encouraging.”*

#### 3.1.3. Facilitators

Mothers described different facilitators that assisted them in the continuation of breastfeeding during the COVID-19 pandemic. The lockdown itself was described as a facilitator for breastfeeding because mothers and their infants were together all the time. For example, one mother explained that she stayed at home because her fear of COVID-19 during the lockdown encouraged her to practice breastfeeding: *“Breastfeeding was easier because of being together all the time”*.

Most participating mothers, especially those who were working, emphasized distance working as a major facilitator of breastfeeding on demand because the mother and her infant were together during distance working hours. For example, one of the mothers stated, *“If there was no COVID-19 pandemic, I would not have thought of breastfeeding at all”*. Moreover, the mothers have recognized that being with the infant almost constantly has encouraged breastfeeding on demand, with no need for pumping or storage.

Additional facilitators for breastfeeding reported by mothers included sharing their experiences about breastfeeding, maternal knowledge, and having the intention to breastfeed. One of the participants described being in a group as a facilitator for the continuation of breastfeeding. She stated, *“We were a group of women, about 15... we all encouraged each other to breastfeed”*.

#### 3.1.4. Challenges to Breastfeeding

Non-working mothers and mothers involved in distance working tended to believe that there were no challenges strong enough to prevent breastfeeding, given that mothers and their infants were together all the time during the lockdown period. However, working mothers in other physical environments during COVID-19 reported different perspectives about breastfeeding.

Working mothers, especially those in healthcare settings, described long working hours as a challenge to the continuation of breastfeeding. One of the working mothers stated, *“The main factor that made me stop breastfeeding is my work”*. For working mothers, it seemed that short break times for breastfeeding added to breastfeeding challenges. One of the participants added, *“We only have one hour … which is not enough for the mother to complete this breastfeeding journey”*. Furthermore, the lack of private spaces or lactation rooms was mentioned by the mothers as a challenge to breastfeeding: *“I have to find a place or bathroom or a prayer room to … breastfeed my child”*.

## 4. Discussion

This study was conducted to explore mothers’ experiences with breastfeeding during COVID-19. Eighteen mothers were enrolled in this study, and based on the data analysis, four main themes emerged. Demographically, most of our participants were working—this is unique among the studies that have been conducted on breastfeeding mothers in KSA. Ahmed and Salih [13] recruited 60% of the mothers in their study from the western region of KSA, where most were housewives. This demographic provides a possible explanation for the data collection and recruitment method that Ahmed and Salih used in their study. Social media was used in this study to recruit the mothers, which might have targeted a specific sample different from the sample recruited in the other study from the primary healthcare centers. The study design could be another factor; our study was qualitative, whereas Ahmed and Salih [13] conducted a quantitative study.

In our study, the duration of breastfeeding for previous babies was 3–22 months, and for the current baby, the duration ranged between 2 and 19 months. These results are consistent with the study that was conducted by Ahmad et al. [22]. Their participants reported that the duration of breastfeeding ranged between 2 months and 3 years.

Additionally, we identified four main themes. The first theme was the breastfeeding experience (including positive and negative experiences). The positive experiences included improved bonding between the newborns and their mothers, the mothers’ beliefs about the importance of breastfeeding for improving newborn immunity, and the mothers’ reports of an easier experience because they did not need to prepare for bottle feedings. These results are consistent with the findings of Wen et al. [23], indicating that mothers perceived breastfeeding as clean, safe, and beneficial for their newborns. Even though these two previous studies had similar findings to the current study, they did not seek to explore the mothers’ experiences with breastfeeding during COVID-19.

In addition to the positive reactions to breastfeeding, some mothers also reported some negative perceptions, including difficulty with breastfeeding, the required commitment, occasional pain, the required effort, and worry about the possibility of transmitting the coronavirus to their newborn during breastfeeding. Brown and Shenker [3] found similar findings: In their study, 80% of the mothers reported being worried about the health of their children if they continued breastfeeding. In order to decrease the worry among mothers, it is important to reassure and educate breastfeeding mothers about the nature of the disease and the modes of transition. This reassurance can come from healthcare providers before discharge from the hospital or during the postpartum and well-baby visits. In addition, in the same study, Brown and Shenker [3] found the mothers mentioned that pain and exhaustion during breastfeeding were reasons to stop. To help mothers overcome the pain and difficulty in breastfeeding, breastfeeding education, including position during breastfeeding, storage of breastmilk, correct latching, etc., should be provided to the mother before birth, and it should be repeated to enforce the saturation of the information in the postnatal period.

The second theme in our study covered support and resources. The mothers reported that the support they received from their husbands, family members, friends, healthcare workers, and social media helped them continue breastfeeding during the pandemic. These results were similar to the results of Wen et al. [23], who found that the motivation came from family members and was based on the cultural norms and the family’s point of view about breastfeeding. In addition, the support from healthcare providers helped the mothers succeed in their breastfeeding. Piankusol et al. [24] found that 60% of mothers continued breastfeeding during the lockdown because of support from their partner, and 93% continued breastfeeding because of the availability of health provider support during the lockdown.

The third theme covered facilitators during the COVID-19 pandemic that helped the mothers initiate and continue breastfeeding. These facilitators included distance working, the lockdown period, the mother’s intention, and the mother’s knowledge about breastfeeding, as well as shared experiences about breastfeeding among women who breastfed during the same period. Social media helped mothers learn more about the best way to deal with breastfeeding during the pandemic, even if a mother contracted COVID-19. The mothers reported that the accounts of breastfeeding educators on Instagram and Snapchat helped them increase their knowledge of breastfeeding and helped them continue breastfeeding for longer periods. The result from this theme can be helpful for breastfeeding mothers if the stakeholders give the mothers the choice to work remotely or have flexible working hours, which will help in the continuity of breastfeeding.

Similar results were found by Brown and Shenker [3]. They reported that 41% of participants found the lockdown to have a positive effect on breastfeeding, whereas 29.5% were neutral regarding the relationship between breastfeeding and the lockdown. A possible explanation for the positive effect in our results and those of Brown and Shenker is that the mothers during the lockdown did not have any social commitments, nor were there any places of entertainment they could go to outside their homes. Even when working from home, which helped the mothers be closer to their children, they might have not needed to use the breast pump frequently.

Additionally, Costantini et al. [25] found that 62% of mothers used the internet, and 52% turned to experienced family members and friends as their sources of information during COVID-19 to help them with their breastfeeding experiences. These findings are similar to the findings of the current study, that social media and shared experiences were facilitators of breastfeeding. The other facilitators in our study were the mother’s intention to continue breastfeeding and the mother’s knowledge of the benefits of breastfeeding. These findings are similar to the study results of Costantini et al. [25]. They found that the majority of mothers strongly committed to continuing breastfeeding during the pandemic because it would protect their infants from contracting the virus or any other infection. Most of the mothers did not choose to wean their infants from breastfeeding during COVID-19.

The last theme in our study concerned the challenges of continuing breastfeeding during the pandemic. The challenges reported by the participating mothers include long working hours (especially for healthcare providers) and a lack of facilities where the mothers could pump and store their breast milk. Ickes et al. [26] interviewed 21 healthcare providers to investigate their experiences with breastfeeding during their working hours. Their results showed that using a breast pump was challenging because of the lack of suitable rooms in which to pump or refrigerators to store the milk. In addition, unviable onsite daycare made it harder for the mothers to provide exclusive breastfeeding to their infants. Even though Ickes et al. [26] did not focus specifically on breastfeeding during COVID-19, the researchers provided several suggestions to improve the breastfeeding experiences for working mothers—especially healthcare providers who work for long periods during the day. These suggestions included decreasing working hours and providing onsite daycare. However, these were not viable choices for mothers during the pandemic because most of the healthcare facilities did not allow any persons other than patients to enter the facilities to decrease the chances of transmitting the coronavirus. The other recommendations to improve the exclusive breastfeeding experience were to increase the length of maternity leave and provide a lactation room containing the necessary equipment and a refrigerator.

## 5. Conclusions

Although the participants were varied in their sociodemographic status, most of them were from the western region, which may limit the transferability of the study results. Moreover, the results depicted the experiences of breastfeeding mothers during the COVID-19 pandemic and were not specifically focused on representing the experiences of breastfeeding while the mothers or their infants were infected with COVID-19.

The results of this study highlighted the mothers’ experiences with breastfeeding during COVID-19. Future researchers may use the results of this study to reinforce the positive experience of breastfeeding to strengthen the bonding between mothers and infants during the pandemic. Considering that some mothers in this study were worried about becoming infected,

The results of this study revealed four themes: experience, support, facilitators, and the challenges of breastfeeding during the COVID-19 pandemic. Most mothers depicted their experiences with breastfeeding during the pandemic as an encouragement to continue breastfeeding and enhancing the mother–newborn bonding. However, some mothers were worried about contracting COVID-19 and transmitting it to their newborns. Support from family members during breastfeeding was found to play a role in the continuation of breastfeeding during the pandemic.

Future research is needed to understand the experience of breastfeeding among infected infants of mothers who test positive for COVID-19. The findings from this study lay the foundation for future research to support breastfeeding practices and overcome the challenges that occurred during the pandemic.

## Figures and Tables

**Table 1:** Sociodemographic characteristics.

Characteristics		*n* (%) Range
Obstetric History	Number of pregnancies	Range (1–5)
	1	5 (28%)
	2	6 (31%)
	3	2 (11%)
	4	4 (22%)
	5	1 (6%)
	Number of deliveries	Range (1–3)
	1	5 (28%)
	2	7 (39%)
	3	6 (33%)
	Number of abortions	Range (0–2)
	0	11 (61%)
	1	6 (33%)
	2	1 (6%)
Children	Number of living children	Range (1–3)
	1	5 (28%)
	2	7 (39%)
	3	6 (33%)
	Age of current child	Range (2–19 months)
Breastfeeding and Skin-to-skin contact	Previous breastfeeding	
	Yes	13 (72%)
	Not applicable (first pregnancy)	5 (28%)
	Length of previous breastfeeding	Range (3–22 months)
	Length of current breastfeeding	Range (2–19 months)
	Lactation consultant visit	
	Yes	13 (72%)
	No	5 (28%)
	Skin-to-skin contact after labor	
	Yes	12 (67%)
	No	6 (33%)
COVID-19	Mother infected with COVID-19 during breastfeeding period	
	Yes	2 (11%)
	No	16 (89%)
	Family member infected with COVID-19 during breastfeeding period	
	Yes	12 (67%)
	No	6 (33%)

**Table 2:** Findings on breastfeeding themes during COVID-19.

Breastfeeding during COVID-19	
***Positive***	***Negative***
1. Bonding between the mother and the newborn 2. Encouraged to strengthen newborn immunity 3. Easier (no need for pumping or preparing bottle feedings)	1. Not easy 2. Commitment 3. Pain 4. Effort 5. Worries/fear of contracting COVID-19
**Support/Resources**	
1. Family members (husbands, mothers) 2. Friends colleagues 3. Hospital support (lactation consultant, baby-friendly hospitals) 4. Social media	
**Facilitators**	
1. Distance working 2. Sharing experiences about breastfeeding 3. Mother’s knowledge of breastfeeding benefits 4. Being with the baby all the time (during lockdown period) 5. Mother’s intention to breastfeed	
**Challenges**	
1. Long working hours (healthcare sector) 2. No facilities available (breastfeeding rooms)	

## Data Availability

The data are available from the authors on request.
